# The Concerns of Competent Novices during a Mentoring Year

**DOI:** 10.1155/2012/812542

**Published:** 2012-06-03

**Authors:** Susan Lennox, Annemarie Jutel, Maralyn Foureur

**Affiliations:** ^1^Graduate School of Nursing, Midwifery and Health, Victoria University of Wellington, P.O. Box 7625, Wellington 6140, New Zealand; ^2^Centre for Midwifery, Child and Family Health, Faculty of Nursing, Midwifery and Health, University of Technology, Sydney, Broadway, NSW 2007, Australia

## Abstract

In an innovative group mentoring approach, four experienced midwives mentored four new graduates during their first year of practice. The new graduates were in practice as case-loading registered midwives having completed a three year Bachelor of Midwifery degree. Detailed data about the new graduates' concerns were collected throughout the year of the mentoring project. A range of practice areas—administrative, working environment, professional culture, clinical issues and the mentor group itself—were prominent issues. New graduates were concerned about their own professional development and about relationships with others particularly relationships within the hospital. Technical questions focussed more on craft knowledge that develops through experience than on clinical skills or knowledge. Identifying these concerns provides a foundation for mentors, preceptors and those designing professional development support programmes for the first year of practice. It may be that new graduate midwives educated in a profession with a narrowly defined scope of practice have a different range of concerns to new graduates who have wider scopes of practice. The use of a group model of mentoring for supporting new graduate midwives proved stimulating for mentors and highly supportive of new graduates.

## 1. Introduction

Mentoring or preceptoring in the first year of practice in nursing and midwifery has nearly always been thought of as a one-to-one relationship in which an experienced practitioner supports a novice [1]. By contrast, this paper is derived from a naturalistic study of an innovative approach in which a group of experienced midwives worked together to mentor a group of new graduates [2]. The study was based on an assumption that the new graduates were competent novices who wanted support to develop their confidence for practice. The new graduates were responsible for seeking the support they needed and for raising concerns for discussion in regular group meetings with the mentors. Therefore, this approach to mentoring rested on adult learning principles where the learners' identified their individual concerns as they arose. This paper describes their concerns. It will be of interest to mentors and preceptors in nursing and midwifery whether they wish to work as a group or in the more usual one-to-one relationship.

## 2. Background: What This Paper Is about and Why It Is Important

The midwives who were mentored in this study had just graduated from a three-year Bachelor's degree in midwifery (without prior registration as a nurse) and were newly registered as midwives. Practising certificates issued annually by the Midwifery Council of New Zealand are based on competency as an entry level practitioner [3]. In New Zealand, midwives called lead maternity carers (LMCs) can provide total care to women throughout the childbearing experience from diagnosis of pregnancy, through the intrapartum experience and followup to six weeks after the birth. This primary care service is government funded, and midwives can elect to work as self-employed case-loading practitioners from the first year of registration.

In 2006, four final year midwifery students were planning their next year as autonomous practitioners and seeking one-to-one mentoring. They had chosen to become LMC midwives case-loading as self-employed midwives. The four new graduates were planning to work together in a group practice. At this time there was a workforce shortage in midwifery, and mentor midwives were in short supply [4]. As a response to the students' practice need, four experienced mentors, who could not provide around the clock one-to-one support, agreed to share the mentoring responsibility within a group [2]. The resultant group consisted of four new graduates and four experienced midwives.

A group mentoring model was coconstructed with input from the students and the mentors, and a contract was negotiated prior to the commencement of the graduates' first year. The purpose of the group mentoring initiative was to support the development of confidence in the graduates. The starting point for the project was that new graduate midwives were competent novices who could identify what they needed to develop practice confidence. Therefore, the group mentoring approach was strongly centred on responding to new graduates' self-perceived concerns rather than imposing a professional curriculum during their transition to practice.

Concomitantly, all of the members of this group mentoring project agreed to participate in a research project designed to capture the novelty and efficacy of the approach, its strengths and weaknesses. The research involved a detailed analysis of the concerns identified by the new practitioners and how these varied over the course of the year. This paper reports on that component of the research and specifically on mentoring rather than new graduate literature. As recently identified, knowledge about how best to support midwives is sorely needed [5]. Evidence has accumulated about the transition to practice which exposes the first year in practice as challenging [6, 7]. The context in which these concerns were expressed was through group mentoring which is an unusual approach for professional support in the first year of practice. Mentoring has traditionally been thought of as a one-to-one relationship.

### 2.1. History and Concept of Mentoring

Historically, mentoring emerged out of antiquity from the works of Homer, and in particular the *Odyssey* [8]. When Odysseus left for war he entrusted the care of his only son, Telemarchus, to his friend, Mentor. Later when the goddess Athena visited the young adult Telemarchus dressed as a man, she did so to “embolden him.” Contemporary mentorships emulate this classical tale where one adult is more experienced than another in some aspect of their career. Mentoring occurs during professional transitions such as emerging from training (new graduate) or when there are significant changes in career circumstances.

In the 1960s, mentoring emerged in the United States as a very successful career development relationship [9, 10]. Kram, a business management researcher, analysed 18 mentoring relationships and described the functions that mentoring served [11]. These were divided into two major categories: instrumental and psychosocial and nine subfunctions. This analysis has stood the test of time, and the functions have since been used as the benchmark for many studies.

Nursing soon saw the benefits of mentoring and Yoder [12], a nurse researcher, created a concept analysis of how mentoring operated across the disciplines of business, education, and nursing. Vance [13], a leader in nursing, identified mentoring as useful for women in leadership but also envisioned a time when this support could be more universally available and “evolve into some form of institutional support in many organizations” [13]. Vance's encouragement to nurses suggests that both the mentor and the mentee gain from the experience of mentoring, giving some credence to the notion that the female developmental journey “emphasize[s] connection and care” [14].

### 2.2. Mentoring as a Developmental Process

Models of mentoring characterise how power is expressed in the relationships. A developmental model of mentoring is characterised by the mentee taking the active role in the relationship rather than the mentor so that “empowerment and personal accountability” are emphasized [15]. Developmental mentoring is a partnership established with an end purpose in mind, such as encouraging confidence in a particular occupation or position or at a particular stage, such as the first year in practice. The plans and processes for achieving this end are purposely put in place by mutual dialogue and negotiation. Both parties are engaged in the process of achieving this end without the mentor using their influence to privilege the mentee. The purpose of the mentoring relationship is to enhance the mentee's development by inspiring the mentee to a greater understanding of the role. The learning process is shared: the mentee is learning about a role or increasing expertise, and the mentor is learning about the process of stimulating developmental changes. In New Zealand, this form of mentoring resonates with the partnership model of midwifery, where, as the primary maternity providers, midwives actively encourage women's choices and shared responsibility [16, 17].

### 2.3. How Group Mentoring Operated

Mentoring was defined in this study as “a voluntarily agreed professional support activity in which the person being mentored is the active partner, their needs are the focus of the mentoring, and the mentor's intention is to assist and cultivate their professional confidence” [2]. Meeting the new graduates' needs by ensuring the new graduates take the active role defined the mentoring relationship. In such a relationship, the “less experienced person (mentee) aims to gain knowledge, develop skills, and achieve insights with the help of the more experienced person (mentor)” [18]. The purpose of the relationship was to develop new graduate confidence, a purpose which is in line with the NZCOM consensus statement on mentoring and which informed the contract the group initiated and developed [19].

The terms of the group mentoring project were that the new graduates were able to contact a mentor at any time, 24 hours a day over the whole year. Group meetings were held weekly for the first eight months and then fortnightly and finally every three weeks for the remainder of the year. Attendance was voluntarily, but few meetings were missed by the new graduates, and there was only one meeting out of 31 when only one of the four mentors attended. The number of meetings and the length and the structure of the process were all negotiated between members of the group. The meetings generally took two hours and were facilitated by each of the eight participants. The meetings followed a structure which was designed to enable the new graduates to bring up their concerns and for these to be the focus of every meeting.

## 3. Method: How New Graduates' Concerns Were Identified

### 3.1. Theoretical Underpinning and an Overview of Data Sources and Analysis

The theoretical underpinning to the research was pragmatic mixed methods, accommodating the values and limitations of quantitative and qualitative paradigms [20]. Pragmatism as a philosophy emerged in the early twentieth century and challenged the hitherto belief that knowledge could only be generated by thinking. Hume contended that matter or even the world out there was no more than a “very useful hypothesis” [21].

Therefore, Dewey's contention that knowledge was generated by action and reflection, where mind and matter were connected, was a distinctly different philosophy to the sceptical approach borne of dualism where mind existed but matter was beyond the veil of certain knowledge. Dewey himself described his new theory of knowledge as equivalent to the discoveries of Copernicus where the sun rather than the earth was the centre of the known universe [22]. Dewey's “Copernican turn” was to propose a theory of knowing which arose from interacting with the world rather than arising only from the mind. He proposed that knowledge was borne of interactions between (our) actions and (their) consequences and later described these connections between mind and matter, as transactions. Knowledge from research transactions was, he maintained, reconstructions of our experiences and had the status of warranted assertions but that did not make them certainties. Real experience should therefore not be confused with truth claims as real is contextual and temporal knowing. Pragmatism is defined as an approach “that debunks concepts such as ‘truth' and ‘reality' and focuses instead on ‘what works' as the truth regarding the research question under investigation” [23].

The approach used in this study was naturalistic inquiry by a participant-researcher who was open to what might emerge from what was an untried practice innovation. It was therefore important that the researcher collect the data without trying to influence the process. Such an approach required that data be collected opportunistically and unobtrusively alongside the mentoring. Both qualitative and quantitative data were collected: recordings of the regular, formal meetings between the new graduates and the mentors; semistructured interviews of each of the eight participants at the beginning, middle, and end of the mentoring year (24 in total); logs of telephone, text or face-to-face contacts between the new graduates and mentors; visual analogue scales of confidence completed by the new graduates during their interviews. The birth data of women cared for by the new graduates was also collected.

The methods of analysis varied for each type of data. The 24 individual participant interviews were recorded, transcribed, and analysed for common themes [2]. A sample of 19 recordings of group meetings were transcribed and analysed using an iterative process to discover points of interest inductively and intuitively, and this resulted in two levels of thematic analysis. The 85 on-call contact logs were analysed using simple descriptive analysis of the number and type of contacts, the reasons contacts were made, and the distribution of the different categories of reasons over the course of the mentoring year.

### 3.2. On-Call Logs

The new graduates chose when to contact mentors for one-on-one support so these contacts reflect their self-identified needs. Therefore, the on-call logs are one source for understanding graduates' concerns. However, since these were completed by the mentors, these are not a primary source, rather they represent the mentors' understanding of the new graduates' concerns.

### 3.3. Weekly Meeting Transcripts

The weekly group meetings were structured around new graduates' concerns, so analyses of the transcribed recordings are the key primary source of data. All 31 of the group meetings were recorded and of these, 19 were chosen from across the year for analysis. The transcripts were the subject of many rounds of iterative thematic analysis finally settling on two levels of themes. The first level themes related to the location, within their scope of practice, of the new graduates' concerns. The second level of thematic analysis looked at 95 threads of conversations in the meetings and identified what concerns prompted each thread, resulting in three primary themes.

## 4. Results and Discussion: New Graduate Midwives' Concerns

This section presents the concerns that led new graduates to make contact with the on-call mentor and then follows with the concerns discussed in the weekly group meetings; initially identifying in which areas of practice concerns were located and then what sorts of concerns prompted discussions.

### 4.1. Concerns That Prompted New Graduates to Contact a Mentor

During the year (January to December), mentors recorded 85 contacts with new graduates: 56 contacts (66%) were phone calls and five (6%) were text messages, on eight occasions (9%) the mentor and midwife met without seeing the client and on 16 occasions (19%) they met together with the client.

**Figure 1:**
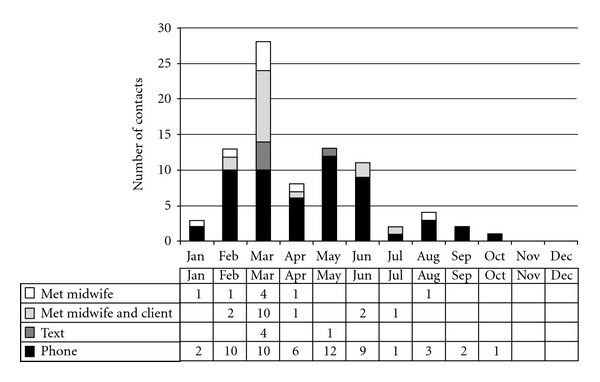
Number, type, and frequency of contacts between mentors and new graduates over one year.

As shown in Figure 1, most contacts occurred in the first six months with only nine contacts from July onwards. The last contact was a single call in October. Of the 16 contacts that involved the mentor being with the new graduate and her client (mostly at a birth), ten (62%) occurred in March (mid-February was the time when the first women cared for by the new graduates started to give birth). On average there were 3.1 contacts (2.6 by phone) for each of the weeks when there were contacts, with the busiest week of the year having 17 contacts recorded (including 4 texts and 5 phone calls).

Mentors recorded a brief description of the reason for each contact. As shown in Table 1, these descriptions were found to fall into one of the following categories: advice, assistance, giving information, discussion, or were initiated by a mentor. “Advice” refers to a simple request for information. “Assistance” refers to a request by the new graduate for backup from the mentor (usually to attend a birth). “Giving information” indicated that the new graduate was providing something to the mentor, often keeping her updated about a client. “Discussion” refers to times where the new graduate wanted to be able to review a situation and talk about her thoughts without needing advice or assistance. On one occasion, a mentor initiated contact by phoning a new graduate to ask about a client's progress.

In summary, mentors were contacted by new graduates several times a week in the first half of the year, but there was considerable variation between new graduates in the numbers and types of contact they initiated. Two-thirds of the contacts involved only a phone call for advice or information, while about a fifth involved the mentor meeting with the new graduate and her client (usually at a birth) and providing assistance. In the second six months, there were far fewer calls, and when they did occur, a greater proportion were contacts where the new graduate was seeking a discussion rather than asking for information, advice, or assistance.

### 4.2. Concerns Raised at Meetings

Five areas of concern were identified from the meeting data: administrative issues, working environment, group culture, professional culture, and clinical issues. These categories were present throughout the year with varying frequency.

Administrative issues were a varied and loose grouping of general administrative matters. The areas covered included questioning the need to document phone calls, problems with hospital access agreements, creating business cards, how to obtain letterhead stationery, and collating email addresses. Such administrative issues were mostly dealt with quickly and did not lead to much discussion. Early in the mentoring year, the new graduates asked simple information gathering questions; but after the first eight meetings, when administrative issues arose, they did so from discussions around practice issues. The change from simple information gathering to practice discussions was swift. For example, in the first meeting, there were 30 such simple information gathering questions, but by the eighth meeting there was only one.

The second area concerned the working environment, and included exchanges relating to the new graduates' work in both the community and the hospital. These concerns included their relationships with others as well as their understanding of how the systems worked in both environments. There was evidence of questioning the place of the midwife within the system, how that accorded with the regulations, and about the bases for on-going collegial relationships. For example, one new graduate reported.

I went in with the bloods [referring to laboratory reports] and said—he said “we need to induce”, I said “why?” and we talked about it. [He] rang the consultant and she said the same (NG4, 14th meeting). (to protect participant identity, new graduates are described as NG, and mentors as M with a unique number to differentiate between the participants in each group).

A conversation then developed with the mentors and new graduates around the management of negotiated conversations between the medical staff, the woman, and her LMC midwife. 

The third concern was group culture and included exchanges about how the mentoring group itself worked, for example, which mentor was on call and who was facilitating the meeting. The group mentoring process unfolded naturally, enabling the new graduates to have as much decision making and facilitative power as the mentors. The new graduates and mentors took turns facilitating meetings and directing the process. Sharing facilitation between the mentors and the new graduates enabled the new graduates to assume power within the group process from the beginning of the group mentoring meetings. The new graduates showed that they felt comfortable critiquing whether the mentoring was functioning well or not, and, therefore, how effectively supported they were by the arrangements in place. The following quote illustrates the new graduates raising an issue reasonably early on in the year about improving access to mentor support:

Three in labour and needing support does not work; because we have no process about a second [mentor] on call (NG 4, 8th meeting).

Professional culture, which was the fourth area identified, entailed discussions about what it meant to be a midwife. This included, for example, being a professional in general, or fulfilling the regulatory bodies' requirements, such as the Midwifery Council's requirements for an Annual Practising Certificate, or attending the NZCOM local meetings, or how the national standards for practice or code of practice were played out in practice. Professional issues were frequently mentioned and discussed, as the new graduates began developing a sense of being a professional and adjusting to their new environment. The range of professional issues is vast and requires the midwife to develop a professional persona.

The clinical aspects of providing care to women did figure in the concerns of the new midwives but was not in any way the dominant focus. For example, one new graduate was talking about a woman for whom she was the lead carer whose baby was presenting by the breech in labour. She sought advice from a specialist obstetrician:

I asked about ECV [external cephalic version] and vaginal birth and [was] told [the] risks [were] too high. If I'd known before she went into labour and she had decided to have a vaginal birth [I would have organised an ECV] (NG2, 14th meeting).

She wanted to critically reflect on the effect this had on the woman and what she and her mentors perceived as her responsibility and not particularly about the evidence about ECV.

### 4.3. What Sort of Situations Prompted New Graduate to Discuss Concerns at Meetings?

For the second level of analysis, the threads of discussion between the new graduates and mentors were examined. The five first level categories were established using mostly isolated quotes from the new graduates, and focusing on the scope and the role of a midwife. Often the reason why an issue was raised did not become obvious immediately but was clearer in the course of the ensuing discussion. For this reason, threads of conversations were used, as exemplified in Table 2.

Each thread began with a new graduate mentioning an issue or question that they wanted to discuss. The thread of the conversation that followed formed the base of the analysis, with contributions from new graduates and mentors. Across 10 meetings, 95 such threads of conversation were identified and coded according to their content. Initially this resulted in identifying ten subthemes. Through a further reading of the material and an iterative coding process, the ten subthemes were grouped into three broad themes: self-reflection, issues to do with others, and technical issues. Of the 95 threads of conversation, 25 were coded as self-reflection, 31 as issues to do with others, and 39 as technical issues. Frequency of a theme is not necessarily indicative of its significance. Each of these three themes is discussed below with examples.

*Self-reflection* involved matters such as reflecting on inexperience, reviewing, and appraising one's own practice, sharing achievements and failures. As the year began, a comment from a new graduate that she “was trying to be confident on the phone” but that she felt “like a fraud” and thinking that the woman, “should ring someone else” preferably “a real midwife” (NG1, 1st meeting). Although the new graduates gained confidence throughout the year, each new experience such as; “I hadn't seen people under a GA [general anaesthetic]” (NG2, 20th meeting) had to be integrated into their understanding, so that the learning became part of their midwife repertoire.

Their level of comfort in this new work world was an insecure one of knowing some things, but being always aware that they would meet yet another new experience. This, one graduate said, was “really hard—[you] lose confidence constantly, feel as though you have to pick yourself up and you do—then you do learn!” (NG4, 20th meeting).

Learning to be assertive was also a constant challenge as new graduates confronted criticism or a sense of being discounted. In the next example, a registrar (a senior doctor in specialist training) wanted to induce labour in a woman late in the afternoon when it was not urgent, and when neither the midwife nor the woman had slept.

This time I need to do what is good for us…I felt last time I got over-ridden and I thought “no, I have to do what is good for us” (NG1, 14th meeting).

The new graduate had met the situation before and knew now that the hospital protocol supported her resistance to a rushed induction, so she had a reasoned argument for not being “over-ridden” this time.

*Issues to do with others *was the second main theme. This included issues such as client emotions, new graduate peer support, observing how others practice, and negotiating the “pecking order” in the institution. There was often a tension between how the new graduates perceived themselves as autonomous practitioners and how others responded to them. Many issues arose from this tension or other aspects of their relationship with others—including other professionals and peers as well as their clients and their families. Many of these issues to do with others were related to the new graduate's autonomy and agency, such as whether they were able to have a voice, show confidence or be silenced, their concern for women, babies and the family, finding the boundaries of professional practice, establishing networks of peers, mentors, staff midwives, coordinators, and other LMCs.

New graduates sometimes found clients' emotional responses challenging because they were in the midst of managing their own emotions and therefore found emotions in others unexpectedly upsetting.

She thought she was going to die; she was so distressed I felt I had to stay; I took the baby out to dad. They were overwhelmed and happy (NG2, 20th meeting).

The new graduates were learning about the emotional work of a midwife, whether this was during labour or during antenatal visits or over the four to six weeks of funded postnatal visits. Whilst one mother remained in hospital, her family cared for the baby at home.

I have been doing the follow up care; baby at home, lots of paranoia, her mother is looking after baby with a mask on, they are very scared (NG3, 29th meeting).

Sometimes events happen about which the family is especially happy. In another case, a woman had a vaginal birth where usually she would have had a caesarean section for a breech presentation, because the breech was undiagnosed and it was too late for a caesarean section.

She said I am so glad I did not have a caesarean section and the husband said he was so pleased she wasn't cut (NG3, 22nd meeting).

The new graduates were very affected by their clients' feelings and although they often shared their observations with the group, they did not appear to need to be reassured; just telling the stories of their clients' emotions was important to them.

As well as issues to do with clients and their families, issues about peers and other professionals were commonly brought for discussion in the group. They easily shared their worries and concerns and found an enormous source of support from their peers.

We have talked about client visits—we chat to one another and ask one another what the other one thought. It's been good (NG1, 1st meeting).

The new graduate peers were also able to provide cover and take over the work when a colleague was tired. “I went for a rest and [one of the new graduates] took over” (NG3, 29th meeting). Sometimes, however, it was only when the new graduate began to reflect on her week that her need for more support became obvious both to her and to others. “Next time hopefully we will be more supportive and you do not have to get to that point” (NG3, 20th meeting).

The experience of hearing about one another's experiences after the event was important for the peer group even if they had been present at the event. The quality of the reflection after such events changed the depth and quality of the learning. Even more frequently, new graduates talked about how experienced professionals practised—not always in a positive light. For example, after a birth, a new graduate was not sure about whether a small tear around the urethra was something she should stitch or not, and she asked for help from the hospital midwife. As the experienced midwife came in, the woman had a short rapid loss of blood, and the midwife's response was to take over.

So I said can you come and check this out to get a second opinion. As she came in the woman had a bit of a bleed and it was flowing. The fundus was not well contracted so she started rubbing up the fundus and expressed a 100 mls clot, then she [the woman] was ok. “Jasmine” [staff midwife] put up a line, got misoprostol put in and the woman went to recovery. She was really dramatic and the woman was like “wow, what a drama”. I had no idea what to do with this [staff behaviour] (NG4, 25th meeting).

The new graduate (who had been practising independently at this point for 10 months) went on to ask how one manages, not the clinical scenario, but the overly dramatic response by a more experienced and senior midwife. At the meeting she was encouraged to accept this event in the context of her inexperience and how, in asking for a second opinion, there needs to be clarity about what help you want.

The lack of negotiation and discussion, especially when the situation was not urgent, surprised and angered the new graduates, but they were unsure how to manage these experiences as revealed in this account.

I wish I had been strong and next time I feel if it is the same circumstances I will just stand my ground. Can I do that? They weren't listening to me (NG2, 20th meeting).

The new graduates appeared affronted by being treated this way, but persisted in the behaviour they believed was appropriate, and at times this approach worked. “Got Reg [Registrar] to come in and see if we can negotiate this” (NG2, 20th meeting).

The experiences of finding themselves at the bottom of the pecking order created a good deal of discussion by the new graduates. Whilst the “issues to do with others” were varied, they were often about how individuals behaved and, as in many of the examples above, were actually about an unsupportive culture. The new graduates' autonomy and capacity to resist the worst of this unsupportive culture and to promote good professional practices was a matter that was commonly brought up and discussed at the group mentoring meetings.

*Technical issues* was the third main theme and covered matters such as administrative details, clinical know-how, and complexity of clinical and social issues in the community. In the first few meetings in particular, many questions were asked about administrative details, and the new graduates became aware of how much of such detail was lacking, despite their preparation for practice. This lack of awareness about the systems included what equipment was needed for practice and where to find the necessary supplies. The new graduates had all studied pharmacology, but at this point none had written a prescription. One new graduate was talking about the need to prescribe iron, but she was unsure about how much supply should be written on the prescription: “[I am] not sure about supply—like 3 months?” (NG2, 1st meeting).

They needed at this point to make clinical decisions about how long the prescription should be made out for–a decision which was not rule bound. This example reflects their world of uncertainty and complexity at the time of beginning in practice.

Sometimes issues were brought up about clinical concerns which led to a discussion of clinical matters. A baby was admitted to the neonatal unit because the mother had a positive group B streptococcal result early in pregnancy when being cared for by another practitioner; but when the new graduate repeated the screening test at 36 weeks gestation, it was a negative result.

Baby now in NNU [Neo-Natal Unit]—thought baby had Group B strep. I took swabs at 36 weeks and they came back negative. I said “ok, I think if [group B] does not show at 36 weeks then [this group B strep is not a problem] ok” (NG1, 9th meeting).

This response by the new graduate showed that she was not aware of the protocol which states that any positive group B streptococcus result should have the baby treated as being “at risk.” The hospital staff were very annoyed with her advice to the woman, and this left the new graduate very shaky when she arrived at the group meeting—so this issue could also be coded under issues to do with others and self-reflection. These themes are often interconnected, and it was typical for clinical issues to entail interacting with health professionals and gaining insights into those people's emotions as well as their own.

Another example shows a new graduate being skilled at identifying and managing a baby who needed assistance to breathe at birth as well as knowing how to manage a woman with a low haemoglobin measurement.

Baby did not spontaneously breathe so needed bagging, started breathing at two minutes, she responded well and quickly and she latched like a dream, she [the mother] is home now, her haemoglobin is 76 but she declined a blood transfusion (NG2, 14th meeting).

There were times when the new graduates sought help from the hospital staff. The next extract shows a new graduate indicating her desire for respectful collaborative practice relationships with obstetrical staff.

I really try not to get defensive inside myself; I think it is really easy just to get defensive. But I am really aware of the fact that I want these people to be on my side, you know that I can communicate with them and have them on board with me. I have been really aware about building those relationships…I have gone to some of the [antenatal obstetrical] consultations—the woman with the anencephalic baby [a cephalic malformation]—and had discussions with the obstetrician, so they know I am there…that I'm a midwife in the community and that I am proactive about things that I need to be proactive about (NG4, 2nd Interview).

This new graduate understood there was more to communicating than finding the right form or the right words to use, and she sought to collaborate and develop effective professional relationships. The next quote, taken from a group mentoring meeting late in the year, shows a new graduate openly acknowledging the limits to her experience and taking responsibility for exploring management strategies in advance.

In terms of post-dates stuff is it different with a VBAC [vaginal birth after a prior caesarean]? I am just concerned with managing something I haven't dealt with much before. She's 39 weeks (NG 1, 27th meeting).

The confidence scales revealed that the new graduates were quite confident in their practice at this stage, so it is important to see that in this case her confidence is appropriately exercised, and she is acknowledging a lack of knowledge in a particular area.

The next quote also taken from a later meeting, illustrates the new graduates' ability to give context to questions asked during mentoring:

How long is it ok for the head to be on view? I had this birth where all was ok, the baby was tachy [tachycardia] for a while, put the CTG on and then variable decels [decelerations of the fetal heart rate] and recovering well; ARM [artificial rupture of membranes], straw [coloured liquor], old mec [meconium], and the heart recovered, she was 7-8 [cms dilated]. We moved to theatre and I was ok with that, got to fully [dilated], [she] did not want to push. I kept showing them the [CTG] trace and getting it signed and all ok. I told reg [registrar] and [reg agreed] we should allow her to do it, so allowed her to breathe the baby down. I let her do that and I had a peep and baby's head was there, the baby came out with Apgar scores of 4, 6, and 8 (NG 1, 27th meeting).

Here the new graduate is confident and is asking for more guidance and information after reflecting on a case and questioning her clinical decision making.

In summary, this second level of analysis shows the issues that new graduates were likely to bring to the group for discussion. These sometimes involved a need for information arising from technical issues but commonly were matters that had caused them to reflect and question their own performance or the way that they interacted with their clients or other professionals. As demonstrated in the examples, these were often situations that had raised difficult questions or various emotions in the new graduates, and they valued the chance to hear the opinions of their peers and mentors.

It is of interest that within this group mentoring model the graduates' concerns reflect the mentoring functions described by Kram in 1980, even though the present study is about a profession rather than the business world and involved a group rather than one-to-one mentoring [11]. In Kram's study of one-to-one mentoring in the business world, she described two main functions of mentoring: “career or instrumental” and “psychosocial” functions [11]. In the present study the technical matters that the new graduates brought to the meetings can be seen to fit Kram's “instrumental” function, and the other two categories, “issues to do with others” and “reflections about self”, can be aligned with Kram's “psychosocial” function.

## 5. Conclusion

The concerns of the new graduates in this study of group mentoring were as much, if not more, about their relationships both within themselves and with others. The technical concerns, when they were presented, were about the kind of craft knowledge that develops through experience and not commonly about task-based knowledge. This group mentorship project presented an opportunity to explore new graduates' concerns in depth, and, perhaps surprisingly, finding relationships were as important as technical issues. This underlines the significance of the model of support for new graduates and that the purpose of the professional development relationship is established at the start and is clear to both parties.

New graduates need to gain confidence in practice and, therefore, to be accepted as well-educated, responsive and caring individuals capable of asking for help when they need it, that is, as competent novices is an important starting point to providing appropriate support. If the education system produces competent novices, then the professions need career development relationships, like mentoring, which speak to their sufficiency.

Understanding the concerns that competent novices are likely to have is important for mentors and preceptors and for those designing mentoring or preceptor programmes. There is little evidence in the international literature that the actual needs of graduates have been studied and the current study, based on close analysis of new graduates' discussions of their experiences, adds significantly to understanding of the topic [5].

## Figures and Tables

**Table 1:** Reasons new graduates contacted mentors over the year.

Month	Advice	Assistance	Giving information	Discussion	Mentor initiated
January	3	—	—	—	—
February	5	4	3	1	—
March	4	12	11	—	1
April	6	2	—	—	—
May	6	1	3	3	—
June	7	3	1	—	—
July	—	1	—	1	—
August	2	—	—	2	—
September	1	—	—	1	—
October	—	—	—	1	—
November	—	—	—	—	—
December	—	—	—	—	—
Total	34 (40%)	23 (27%)	18 (21%)	9 (11%)	1 (1%)

**Table 2:** Example of a thread of conversation (1st meeting).

Speaker	Speech
NG1	We want to ask a really dumb question.
M1	Good we like dumb questions.
NG1	When we are writing to hospital referring people, who do we refer the woman to? Like this woman has fibroids—who do you refer them to? We were told to refer but not who to.
M2	Do you mean who do I ring or where do I send a referral?
NG2	Where do we refer them to? Is it a particular doctor?
M1	You could ring the hospital and talk to a particular doctor. You could ring the hospital outpatients and ask what they prefer; they need to grade them anyway.
M2	When you write a referral begin the letter with “Dear Doctor, thank you for seeing…and then give the reason for the referral and the past and present history.”
M1	There may be a more personal way of doing it by ringing and talking to the doctor.
M2	It's different if an acute thing.
M3	Is the woman term and do you want her seen within 48 hours but not urgently?
NG2	It is a 3 on the referral guidelines. I will ring outpatients.
M1	Good to get a pad to write it on and fax it so you keep a copy.
NG1	We were taught the format for writing the referrals but I just did not know about where or who to send it to so I'll ring outpatients.
